# Redox Homeostasis in Metabolic Syndrome and Type II Diabetes: Role of Skeletal Muscle and Impact of Gold-Standard Treatments

**DOI:** 10.3390/ijms262110370

**Published:** 2025-10-24

**Authors:** Mia S. Wilkinson, Thomas A. Rollin, Michelle Kuriakose, Roan A. L. Haggerty-Goede, Dalia M. Miller, Kimberly J. Dunham-Snary

**Affiliations:** 1 Department of Medicine, Queen’s University, Kingston, ON K7L 3N6, Canada; 2 Department of Biomedical and Molecular Sciences, Queen’s University, Kingston, ON K7L 3N6, Canada

**Keywords:** type II diabetes, metabolic disease, skeletal muscle, redox homeostasis, diabetes pharmacotherapy

## Abstract

Metabolic syndrome and type II diabetes pose a significant international health burden, with the latter characterized by insulin resistance. Patients must rely on therapies that maintain glucose homeostasis when endogenous systems become dysfunctional. Skeletal muscle, as the largest insulin-sensitive tissue in the body, plays a critical role in maintaining glucose homeostasis. During disease progression, chronic nutrient overload shifts redox balance to a pro-oxidant state, further exacerbating metabolic dysfunction. First-line treatments, such as metformin and insulin, along with newly adopted incretin-based therapies, modulate the redox state of skeletal muscle. This review explores how the redox state of healthy skeletal muscle is altered throughout metabolic disease progression and how these changes contribute to a worsening phenotype. We also highlight how each class of regularly prescribed medications targets redox-sensitive systems in skeletal muscle, identifying literature gaps and areas for future investigation.

## 1. Introduction

Diabetes is a chronic, heterogeneous disorder characterized by sustained hyperglycemia. Distinct from type I diabetes mellitus, type II diabetes mellitus (T2DM) accounts for >90% of all diabetes cases worldwide, representing a significant global health burden [1]. More than 530 million adults are estimated to be living with T2DM, with >80% of cases occurring in low- and middle-income countries [1]. T2DM is a disorder of impaired glucose handling, in which chronic hyperglycemia drives compensatory hyperinsulinemia; these sustained states ultimately lead to pancreatic β-cell exhaustion and eventual atrophy [2]. Defined by both insulin resistance and progressive β-cell dysfunction, T2DM can lead to or exacerbate complications such as retinopathy, neuropathy, and cardiovascular disease (CVD) [3,4]. A meta-analysis has revealed a strong correlation between insulin resistance and CVD risk [5].

Cellular homeostasis relies on a fine-tuned balance between oxidation and reduction (redox) reactions to maintain a physiologic equilibrium essential for energetic processes (Key definitions–Box 1) [6]. Oxidative distress driven by supraphysiologic reactive oxygen species (ROS) production accelerates T2DM pathogenesis, highlighting the critical role of redox imbalance in metabolic diseases [7]. T2DM rarely occurs in isolation and is often accompanied by comorbid abdominal obesity, dyslipidemia, and hypertension—core components of the metabolic syndrome cluster; these factors can further perturb cellular redox homeostasis and predispose to CVD [8].

Box 1 **Important Definitions in Redox Biology.**

Free radical: A molecule or atom with one or more unpaired electrons in its outer shell, making it highly reactive [9].

Non-radical: A molecule or atom without unpaired electrons or that is sharing electrons with another free radical. They are less reactive than free radicals [10].

Oxidative Eustress: The physiological range of oxidant production at which redox signaling is regulated [6].

Oxidative Distress: A pathological state when oxidant production overwhelms antioxidant capacity, causing damage and disruption of redox signaling [6].

Antioxidant/Redox Sink: Any molecule or system that neutralizes reactive species or buffers redox shifts. Antioxidants are the defense that maintain balance against oxidants [6].

Skeletal muscle constitutes ~40% of total body weight [11] and is responsible for >70% of whole body glucose disposal; therefore, muscle insulin sensitivity is essential for maintaining systemic glucose homeostasis [12,13,14]. Skeletal muscle insulin resistance is considered the initiating defect in the development of T2DM, often appearing decades before β-cell failure and overt hyperglycemia manifest [15]. Insulin resistance arises in skeletal muscle from imbalanced cellular bioenergetics, wherein metabolic fuel supply exceeds energy demand [16]. Chronic nutrient overload and the resulting dysregulated metabolism disrupt cellular redox balance, causing oxidative distress in skeletal muscle that contributes to insulin resistance and disease pathogenesis. Numerous pharmaceutical and lifestyle-based interventions are part of the standard of care and are often prescribed in combination for the management of T2DM [17]. Many of these therapies modulate the cellular redox state directly or indirectly to restore skeletal muscle metabolic function and insulin sensitivity.

Traditionally, treatment for T2DM has focused on direct glycemic control, while recent therapeutic advancements also target the cardiovascular and renal systems, as well as metabolic drivers of T2DM pathophysiology [18]; however, these therapies do not directly target skeletal muscle. When dietary and lifestyle modifications are insufficient to maintain target glycemic levels, they are supplemented with the gold-standard treatment for T2DM: metformin [19,20,21,22]. Metformin is a biguanide drug that decreases hepatic glucose production, reduces intestinal glucose absorption, and improves insulin sensitivity in peripheral tissues [22,23,24]. If adequate glycemic control is still not achieved with metformin, second-line therapies may be prescribed in combination. Adjunct T2DM therapies include sodium-glucose transport protein 2 (SGLT2) inhibitors, glucagon-like peptide-1 (GLP-1) receptor agonists and dual GLP-1/GIP agonists, dipeptidyl peptidase-4 (DPP-4) inhibitors, insulin secretagogues, and thiazolidinediones [17,19,20,25]. As β-cell function declines during T2DM progression, insulin resistance in peripheral tissues is paired with insufficient insulin production. At this stage of disease, if glycemic control is not achieved with oral medications and lifestyle changes, an individual will be prescribed basal and/or bolus insulin. Overall, the pharmacological management of T2DM has grown beyond direct glycemic control to include diverse drug classes that can target distinct aspects of T2DM pathophysiology and improve patient outcomes [17,18].

Redox homeostasis is crucial to skeletal muscle function, including contractile function and adaptation to stimuli such as exercise and/or shifting metabolic states. The fine-tuned balance between muscle protein synthesis and degradation that maintains healthy skeletal muscle influences both its mass and function; this balance is intimately linked with redox signaling pathways and muscle metabolism. Disruptions to skeletal muscle homeostasis and the presence of oxidative distress are both present and implicated in T2DM pathogenesis and progression. Many of the pathophysiological mechanisms driving the development and progression of T2DM rely on redox homeostasis. As such, many pharmacotherapies modulate redox homeostasis, both systemically and in a tissue-specific manner, including in skeletal muscle. We aim to discuss how the redox state of healthy skeletal muscle is altered throughout metabolic disease development and progression and how these muscle-specific changes contribute to a worsening phenotype. We also highlight how each class of regularly prescribed medications targets redox-sensitive systems in skeletal muscle, identifying gaps in the literature and areas for future investigation.

## 2. Redox Signaling in Healthy Skeletal Muscle

### 2.1. Physiolgical ROS/RNS and Its Roles in Skeletal Muscle

#### 2.1.1. Introduction to ‘Redox’ Signaling

Redox homeostasis is fundamental to biology, underpinning processes from genomic maintenance to the redox metabolome [26]. Redox signaling mediates dynamic organism–environment interactions and influences disease onset and progression [27]. Studying redox biology often involves ROS—a group of molecules derived from molecular oxygen (O_2_) that play a vital role in cellular signaling events. The term ‘ROS’ refers not to a single molecule but to a collective group of O_2_-derived species that vary widely in chemical reactivity (Table 1) [6]. However, due to practical challenges in discerning specific ROS compounds, the term is often used as an umbrella for this suite of complex signaling molecules in redox biology research. Despite this, the species encompassed by the term are diverse and can be divided into free radicals, such as superoxide (O_2_^•−^) and hydroxyl radical (OH^•^), which contain an unpaired electron in the outer orbital rendering them highly reactive and indiscriminate, and non-radicals such as hydrogen peroxide (H_2_O_2_) [26,28]. H_2_O_2_ is well accepted as the primary ROS signaling molecule due to its relative stability and ability to pass freely through cellular membranes and/or utilize aquaporins for transport [29]. Redox signaling through H_2_O_2_ mainly occurs via reversible oxidation of thiolate groups in target proteins [6,28]. Oxidants may also mediate signaling through reversible methionine oxidation, selenoproteins, oxidation of protein metal centers, and oxidized lipids [6]; ROS as pleiotropic signaling agents has been comprehensively reviewed [6,28].

In addition to ROS, other reactive species, such as reactive nitrogen species (RNS), also participate in redox homeostasis, with notable players including nitric oxide (NO) and peroxynitrite (ONOO^−^) [30]. ROS and RNS both serve as signaling molecules to modulate cellular homeostasis and can also interact with one another; NO is considered an important redox sink, able to react with O_2_^•−^ to form ONOO^−^. Cellular redox balance is maintained through stringent regulation of both oxidant production and expression/activation of key antioxidant pathways [31]. Physiological levels of ROS are denoted as ‘oxidative eustress’, highlighting the importance in key signaling pathways [6,26,29]; conversely, ‘oxidative distress’ describes chronic or excessive oxidant production beyond that required for the physiological maintenance of cellular signaling. The updated terms differentiate physiologic and pathogenic redox signaling better than the widely adopted term ‘oxidative stress’ [6].

**Table 1:** Properties of key reactive oxygen and nitrogen species.

ROS/RNS Molecule	Generated by	Reactivity * (L mol^−1^ s^−1^) [32]	Diffusion Capacity/Estimated Distance	Signaling Specificity	Half-Lives (Approximate)	References
**Hydrogen Peroxide (H_2_O_2_)**	SOD and oxidase enzymes	2 × 10^−2^	Membrane-permeable and use aquaporins; 100 µm	High	40 ms–2 s	[33,34,35]
**Hydroxyl Radical (OH** **^•^** **)**	H_2_O_2_ reaction with Fe^2+^/Cu^+^ or ONOO^−^ breakdown	7 × 10^9^	Essentially no diffusion, attacks nearest targets; 12 nm	No signaling role	3.5 ns	[36,37]
**Nitric Oxide (NO** **^•^** **)**	NOS enzymes	Very slow	Membrane permeable; 2–190 µm	High	2 ms–2 s	[36,38]
**Peroxynitrite (ONOO^−^)**	Reaction of O_2_^•−^ with NO	2 × 10^−1^	Limited diffusion; 96 µm	Pathologically high; limited physiological signaling	0.77 s	[36,39]
**Superoxide (O_2_** **^•^** **^−^** **)**	NOX enzymes and mitochondrial respiration leak; one electron reduction of O_2_	<0.3	Poor membrane permeability; 0.8 µm	Low direct signaling, mainly a precursor to H_2_O_2_/ONOO^−^	35 µs	[35,36,40]

* Apparent second-order rate constants for the reaction of the listed oxidant with methionine in water at neutral pH.

#### 2.1.2. Sources of ROS/RNS in Skeletal Muscle

Skeletal muscle is arguably the most plastic tissue in the body, sensitive to factors such as nutritional status, hormonal balance, activity levels, and health or disease state [11]. This tissue exists in a constant, dynamic balance between muscle protein synthesis and muscle protein degradation, which regulates muscle mass and response to various stimuli; shifts in this balance can lead to muscle hypertrophy or muscle atrophy and wasting [41]. In skeletal muscle, ROS play numerous physiological roles, acting as important signaling molecules necessary to maintain normal function (e.g., contraction) and modulate rates of protein synthesis and degradation [42]. It is well known that skeletal muscle is mainly composed of proteins, which, as discussed, are sensitive to reversible and/or non-reversible oxidation by ROS or RNS; this is especially relevant for branched-chain amino acids and sulfur-containing amino acids [43]. Early discoveries revealed oxidative damage and mitochondrial dysfunction in muscle fibers after exhaustive exercise [44]; it is now widely accepted that ROS are essential regulators of signaling pathways that drive muscle adaptations in response to exercise and inactivity-induced muscle wasting [42]. While skeletal muscle contains various cell types, including vascular smooth muscle and endothelial cells, it is composed primarily of muscle fibers, which are thought to be the major contributors to increased ROS production during muscle contraction [45]. Elevated ROS production in skeletal muscle also occurs in response to diverse stimuli such as hormones and growth factors (including insulin), pro-inflammatory cytokines, and physical and environmental factors [30]. Mitochondria were originally highlighted as the primary source of ROS in contracting skeletal muscle [44], with mitochondrial ROS production occurring mainly through ‘electron leak’ from complexes I–III of the electron transport chain (ETC) [46]. However, experimental evidence has shown that the NAD(P)H oxidase (NOX) enzymes are major contributors to ROS generation in contracting muscle fibers [45,47,48]. NOX enzymes exist in three isoforms in skeletal muscle: Nox1, Nox2, and Nox4; with Nox2 and Nox4 implicated in contraction-induced ROS production, insulin signaling and glucose transport, and myocyte response to osmotic stress [49,50]. While mitochondria and NOX enzymes serve as dominant sources of physiological oxidant production, xanthine oxidoreductase (XOR), cyclooxygenases (COXs), and lipoxygenases (LOXs), linked to the activity of the phospholipase A (PLA_2_) enzymes [51], have also been implicated in skeletal muscle ROS production [47].

While ROS are vital to skeletal muscle function, RNS also play important signaling roles in regulating O_2_ uptake and vascular dynamics. The primary enzymes responsible for NO production are nitric oxide synthases (NOSs), which exist in three isoforms: neuronal (nNOS), inducible (iNOS), and endothelial (eNOS) [52]. The predominant isoform in skeletal muscle responsible for NO production is nNOS. NOS enzymes produce NO from L-arginine, O_2_, and NADPH; NOS enzymes may also produce O_2_^•−^ when uncoupled from the substrate L-arginine and cofactor tetrahydrobiopterin [40,53]. NO plays important physiological roles, recognized for its modulation of vascular tone [54], immune regulation [55], and skeletal muscle adaptations to exercise [56]. When NO reacts with O_2_^•−^, highly reactive ONOO^−^ is formed, which can cause DNA fragmentation and lipid oxidation [40]. Skeletal muscle has also been identified as a storage site for nitrate and nitrite, which can be converted to NO by XOR to maintain NO homeostasis under stressed conditions [57,58].

##### Mitochondrial ROS

The mitochondria have long been recognized as a primary site of ROS production in skeletal muscle [42]. Known for performing numerous regulatory and metabolic functions, including maintenance of calcium homeostasis, regulation of apoptosis, and regulation of gene expression, mitochondria are best known for their role in producing cellular energy in the form of ATP via oxidative phosphorylation (OXPHOS) [59]. The mitochondrial electron transport chain (ETC) passes electrons derived from reducing equivalents while simultaneously pumping protons into the intermembrane space, establishing an electrochemical proton gradient that drives the phosphorylation of ADP to ATP at complex V (ATP synthase). During this process, electrons are transferred to O_2_, fully reducing it to water.

Mitochondrial ROS generation primarily occurs through electron leakage, causing the partial reduction of O_2_ to O_2_^•−^, which is rapidly dismutated to H_2_O_2_ [60]. As ROS generation is subject to stringently controlled spatiotemporal regulation, the location of O_2_^•−^ production (mitochondria matrix vs. intermembrane space) determines its signaling potential [61]. While it was historically believed that 2–5% of oxygen consumed by mitochondria is converted to ROS [62,63], more recent evidence indicates that an order of magnitude less (~0.15%) is actually involved in ROS production [42].

##### NOX Enzymes

NOX enzymes are membrane-bound protein complexes, and their main function is the generation of ROS in the form of O_2_^•−^ [49]. NOX enzymes canonically transfer electrons from the donor NADPH to O_2_ to generate ROS extracellularly; however, it has been demonstrated that in adult skeletal muscle, NOX enzymes may preferentially utilize NADH as the electron donor [64]. In cultured skeletal muscle cells, the expression pattern of NOX isoforms is Nox4 > Nox2 > Nox1, but a physiological role for Nox1 in skeletal muscle is not well described [49]. Nox2 has been most heavily investigated as the primary enzyme involved in ROS production during muscle contraction and has also been implicated in the insulin signaling pathway. In response to electrical stimulation, Diaz-Vegas et al. [48] demonstrated increased ROS production in skeletal muscle mediated by extracellular ATP activation of the purinergic receptor P2Y1; they linked this mechanism to Nox2 activity driven by protein kinase C (PKC). Rac1 activation has also been shown to regulate O_2_^•−^ production by Nox2 in skeletal muscle [49]. Work in human and mouse skeletal muscle has shown that cytosolic ROS production by Nox2 regulates glucose uptake during moderate-intensity exercise [65]. In addition to Nox2, Nox4 expression is increased in murine and human skeletal muscle following acute exercise and is associated with increased expression of the antioxidant regulator nuclear factor erythroid 2-related factor (Nrf2) [50]. Knockout of Nox4 revealed that it is required for exercise-induced oxidant production in skeletal muscle.

##### Additional Enzymatic Sources of ROS/RNS in Skeletal Muscle

In addition to mitochondria and NOX enzymes, several other enzymatic systems contribute to ROS/RNS production in skeletal muscle. Oxygenase enzymes, such as COXs and LOXs, generate ROS as byproducts of arachidonic acid metabolism, linking oxidant production to inflammatory signaling [66]. Phospholipase enzymes, particularly PLA_2_, facilitate the liberation of fatty acid substrates that fuel oxygenase activity, indirectly contributing to ROS generation. Finally, NOS enzymes produce NO as a key signaling molecule; however, under conditions of substrate or cofactor limitation, NOS enzymes may become ‘uncoupled’, producing more O_2_^•−^ and thereby amplifying oxidative distress [53]. Together, these additional enzymatic sources complement mitochondrial and NOX-derived oxidants, underscoring the diverse and integrated network of ROS/RNS production in skeletal muscle. Importantly, the signals generated by this network converge on a variety of redox-sensitive systems that govern muscle metabolism, contractility, and adaptation.

##### Redox-Sensitive Systems in Skeletal Muscle

In skeletal muscle, numerous signaling pathways are directly modulated by redox cues, allowing oxidants to act as second messengers that coordinate metabolic and adaptive responses. Established pathways that are redox-regulated include the regulation of cellular kinases [67,68], calcium ion channels [69], and transcription factors [70]. Oxidants such as H_2_O_2_ can activate signaling pathways involving kinase cascades, including mammalian target of rapamycin complex 1 (mTORC1), mitogen-activated kinases (MAPK), AMP-activated kinases (AMPK), phosphoinositide 3-kinase (PI3K)/protein kinase B (Akt), and apoptosis signal-regulating kinase 1 [71]. Redox-sensitive transcription factors include Nrf2, nuclear factor kappa-light-chain-enhancer of activated B cells (NF-κB), hypoxia-inducible factor 1α, activator protein 1 (AP-1), and CCAAT/enhancer-binding protein delta [70]. Oxidants can also activate NAD^+^-dependent deacetylase Sirtuin 1, which in turn regulates Forkhead box class O (FOXO) and p53 transcription factors [28]. The activity of these redox-sensitive systems depends on a finely tuned balance between oxidant generation and antioxidant defenses, providing the foundation for the antioxidant pathways discussed below [26].

### 2.2. Antioxidant Defense Systems

Cellular ROS is controlled through a variety of enzymatic and non-enzymatic antioxidant mechanisms [42]. Primary enzymatic defenses include superoxide dismutase (SOD), glutathione peroxidases (GPX), peroxiredoxins (PRX), and catalase, while non-enzymatic defenses include vitamins C and E [28]. SOD catalyzes the dismutation of O_2_^•−^ to H_2_O_2_ and water, with its three isoforms localized to distinct subcellular compartments (mitochondria, cytoplasm, extracellular space). GPXs, PRXs, and catalase all act as redox sinks for H_2_O_2_. Both PRXs and GPXs exhibit multiple isoforms that localize to different cellular compartments, with PRXs often considered the most important peroxide scavenging enzymes [72]. PRXs reduce H_2_O_2_ through various reactions, generating oxidized PRX that must be reduced by thioredoxin to restore its catalytic activity. GPXs convert H_2_O_2_ to water and alcohols and typically require electrons supplied by reduced glutathione (GSH), which is regenerated from its oxidized form (GSSG) by glutathione reductase using NADPH [73]. Catalase converts H_2_O_2_ to water and is expressed across several cellular compartments, exhibiting one of the highest turnover rates of any enzyme. Mitochondrial nicotinamide nucleotide transhydrogenase (NNT) also contributes to ROS clearance by shifting reducing equivalents from NADH to NADPH [40]. Under homeostatic conditions, ROS are maintained at steady-state levels through finely tuned control of both oxidant production and removal via antioxidant systems (i.e., redox sinks).

Beyond the core enzymatic and non-enzymatic systems, antioxidant defenses in skeletal muscle exhibit remarkable compartmentalization and adaptability. Mitochondrial isoforms of SOD and GPX provide localized protection against respiratory chain-derived oxidants, while extracellular SOD maintains redox balance in the interstitial space [74]. The glutathione and thioredoxin systems also demonstrate crosstalk, ensuring redundancy in H_2_O_2_ detoxification and redox-sensitive signaling [75]. Antioxidant capacity is dynamically regulated by physiological stimuli such as exercise, which enhances PRX and GPX expression, and is diminished under conditions of aging, insulin resistance, or diabetes [76,77,78]. This plasticity underscores the importance of antioxidant systems not only maintaining steady-state ROS levels but also enabling adaptive responses to metabolic and contractile stress.

### 2.3. Insulin Signaling Dependence on Redox-Sensitive Pathways

#### 2.3.1. Overview of Insulin Signaling in Skeletal Muscle

**Figure 1:**
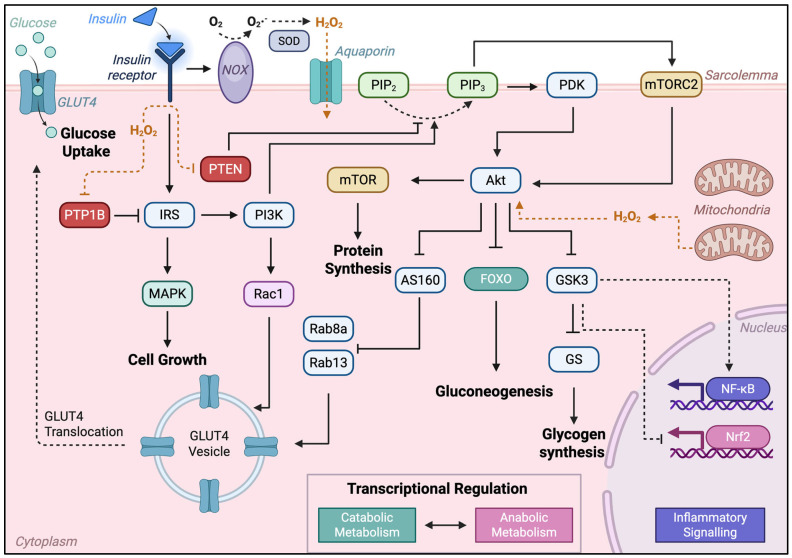
Insulin signaling pathway in skeletal muscle and role of ROS and redox sensitive systems. Insulin binds its receptor leading to activation of IRS and production of O_2_^•−^ via NOX enzymes, which is dismutated by SOD to form H_2_O_2_ that can enter the cell via aquaporins. Mitochondria also produce H_2_O_2_ that participate in the insulin signaling cascade. PTP1B is a negative regulator of insulin, acting through inhibition of IRS phosphorylation. Physiological levels of H_2_O_2_ inhibit PTP1B, thereby augmenting insulin’s downstream actions on PI3K and MAPK. MAPK activation leads to cell proliferation and growth. IRS also activates PI3K which mediates many of insulin’s action on cellular metabolism. PI3K mediates the conversion of PIP_2_ to PIP_3_, which activates PDK and mTORC2 to activate Akt. H_2_O_2_ can also augment activation of Akt at physiological levels. Akt activates mTOR leading to increased protein synthesis and inhibits AS160 and GSK3 which lead to GLUT4 translocation and activation of glycogen synthase (GS) to promote glycogen synthesis. AS160 mediates GLUT4 translocation through its action on Rab8a and Rab13, which is complemented by Rac1 activation by PI3K. GSK3 is also involved in regulation of transcription factors like activation of NF-κB and Nrf2 which are involved in positive regulation of inflammatory signaling and anabolic metabolism, respectively. Akt activation also inhibits FOXO transcription factors, thereby inhibiting catabolic metabolic processes like gluconeogenesis. As shown, physiologic level of ROS are crucial for modulating both negative regulators of the insulin signaling cascade, as well as augmenting activation of key players like Akt and IR phosphorylation. Solid black arrows represent activation or phosphorylation; dashed arrows indicate conversion of molecules or processes involving translocation across the cell; orange arrows denote ROS/redox signaling; blue nodes represent key stages in insulin-dependent signaling; purple, pink, and teal nodes show transcription factors and respective boxes reflect the broad outcomes of this transcriptional regulation; dark red nodes represent negative regulators of insulin signaling.

Insulin signaling in skeletal muscle is a tightly regulated process that underpins glucose uptake, glycogen storage, and metabolic adaptation (Figure 1). It begins with insulin binding the insulin receptor (IR), activating its intracellular tyrosine kinase domain, and inducing tyrosine phosphorylation of insulin receptor substrates (IRS) [79]. Activated IRS proteins recruit PI3K, which hydrolyzes the membrane-bound phosphatidylinositol bisphosphate (PIP_2_) to phosphatidylinositol-3,4,5-trisphosphate (PIP_3_), thereby recruiting phosphoinositide-dependent kinase 1 (PDK1). PDK1 partially activates Akt, with full Akt activation completed by mammalian target of rapamycin Complex 2 (mTORC2, key subunit Rictor). PI3K-Akt activation represents a key signaling node in the insulin pathway, mediating most PI3K-dependent metabolic actions of insulin through phosphorylation of several substrates, including kinases, signaling proteins, and transcription factors [80]. For example, downstream phosphorylation and inactivation of both glycogen synthase kinase 3 (GSK3) and Akt substrate 160 (AS160) promote glycogen synthesis and regulate glucose uptake, respectively [80].

The first described Akt/PKB target was GSK3 [81]; its phosphorylation and inactivation decrease activity toward glycogen synthase, resulting in increased glycogen synthesis. Other protein kinases, such as protein kinase C (PKC), c-AMP-dependent protein kinase A (PKA), and S6 ribosomal protein kinase, can also inhibit GSK3 by phosphorylation; however, Akt is the primary mediator of insulin-dependent GSK3 inhibition. In addition to regulating glycogenesis, GSK3 inhibits insulin-induced glucose transporter 4 (GLUT 4) translocation to the plasma membrane through phosphorylation-dependent inactivation of kinesin, a motor protein involved in GLUT4 membrane trafficking. GSK3 is also implicated in NF-κB activation, thereby contributing to inflammatory signaling.

PI3K activation also represents a critical bifurcation point, activating parallel pathways that are essential for GLUT4 translocation through its effects on both Akt and Rac1 [14]. Inactivation of the Rab-GTPase-activating protein AS160 by Akt triggers activation of Rab small GTPases involved in cytoskeletal organization required for GLUT4 translocation to the plasma membrane—specifically Rab8a and Rab13 in skeletal muscle [82,83]. Simultaneously, PI3K activates the Rho family GTPase Rac1, which drives a cytoskeleton-regulating cascade involving dynamic cortical actin remodeling through the ARP2/3 complex and cofilin; this parallel pathway involves the proto-oncoprotein c-Cbl. The c-Cbl/CAP cascade is an independent yet important pathway that, along with PI3K-Akt, serves as a dominant mediator of insulin-dependent glucose uptake through GLUT4.

Akt also activates the mTOR pathway, regulating protein synthesis, and controls the expression of lipogenic enzymes by modulating the activity of Forkhead box transcription factors. In addition, IRS proteins activate the Ras-MAPK pathway to regulate gene expression and cooperate with the PI3K pathway to control cell growth and differentiation.

PI3K also participates in the negative regulation of insulin signaling via regulatory subunits, which act via multiple mechanisms. The monomeric, noncatalytic p85 subunit can inhibit binding of IRS proteins to the catalytic subunit and may sequester or compartmentalize PI3K activity. Crosstalk between p85 and the stress kinase pathway also regulates PI3K activity and insulin signaling. Notably, the p85 subunit is required for insulin-dependent activation of c-Jun N-terminal kinase (JNK) and has been shown to regulate c-Cbl, Rac1, and cell-division cycle 42. These findings suggest that, beyond regulating the catalytic subunit of PI3K, the regulatory subunit p85 has multiple mechanisms of regulating insulin sensitivity. Another established negative regulator of insulin signaling is protein tyrosine phosphatase 1B (PTP1B), which dephosphorylates the IR kinase domain, PI3K, and Akt [84]. A final notable negative regulator of insulin signaling is phosphatase and tensin homolog (PTEN), which dephosphorylates PIP_3_, thereby disrupting downstream activation of PDK [85].

#### 2.3.2. Redox Actions in the Insulin Signaling Cascade

One of the first connections between insulin and ROS was the observation that H_2_O_2_ can activate insulin signaling or insulin-like metabolic actions [86,87]. This activation was shown to occur through insulin-independent tyrosine phosphorylation of the IR [88]. As discussed, muscle contraction increases intracellular ROS, particularly H_2_O_2_, prompting researchers to examine whether this contributes to meeting the energetic cost of contraction. Literature suggests that H_2_O_2_ elevation during skeletal muscle contraction stimulates glucose uptake in a dose-dependent manner [89]; notably, H_2_O_2_-stimulated glucose uptake is abolished by PI3K inhibition, implicating this pathway in mediating this response. Mechanistic studies indicate that H_2_O_2_ can enhance insulin signaling by promoting tyrosine phosphorylation of IRS proteins and augmenting PI3K–Akt activation [90,91]. While it has been reported that insulin action causes H_2_O_2_ production in skeletal muscle, this notion was recently challenged [92]. Henríquez-Olguin et al. observed no rise in intracellular ROS following insulin stimulation in murine and human skeletal muscle and report a pro-reductive shift in cysteine modifications impacting key insulin signal transduction proteins like GSK3 and Ras [92]. The insulin-mimetic properties of H_2_O_2_ should not be confused with insulin-stimulated H_2_O_2_ production, and the relationship between insulin-induced ROS production and interaction with insulin and its transduction proteins warrants further investigation using contemporary methods and human subjects.

Interactions between ROS and the insulin pathway have been recently reviewed [93]. Briefly, IR activation by insulin binding is thought to activate NOX enzymes to form O_2_^•−^, which is converted to H_2_O_2_ rapidly via SOD; this localized ROS production interferes with inhibitory effects on IR phosphorylation by released ADP, effectively prolonging signaling activity [94]. As described, activation of PI3K-Akt is essential for insulin signal transduction and action, and is negatively regulated by both PTEN [95] and PTP1B [96]; both are inactivated by oxidation from H_2_O_2_. Insulin-stimulated H_2_O_2_ inhibits the negative regulator PTP1B, thereby enhancing insulin’s action and downstream effects [96]. As well as inhibiting negative regulators of insulin action, H_2_O_2_ can act on key cysteine residues in Akt to form a disulfide bond that enhances recruitment to the plasma membrane, thereby increasing Akt activation and positively regulating downstream actions of this key signaling node [97]. In contrast, high levels of ROS can form a second inhibitory disulfide bond in Akt’s kinase domain, reducing its activity [98].

These effects appear to follow a hormetic pattern: physiological or transient increases in ROS amplify insulin sensitivity, whereas chronic or excessive ROS generation disrupts signaling and contributes to insulin resistance [42,99]. Indeed, glucose uptake in C2C12 myotubes and isolated single muscle fibers under oxidative eustress is enhanced [100]. Conversely, excessive oxidation during oxidative distress compromises cell viability and blunts the increase in glucose uptake seen under oxidative eustress conditions. In the context of metabolic disease, sustained oxidative distress has been shown to impair GLUT4 translocation and dysregulate downstream effectors, highlighting the dual role of ROS as both facilitators and inhibitors of insulin action depending on concentration, duration, and cellular context [101,102,103,104].

## 3. Redox Signaling During Metabolic Disease Progression

### 3.1. Redox Damage and Disruptions to Insulin Signaling

**Figure 2:**
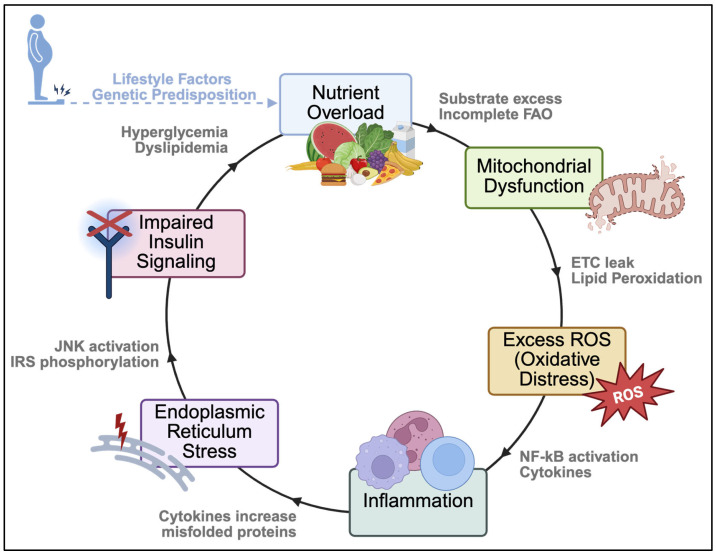
Interconnected cycle of nutrient overload, redox imbalance, and insulin resistance in the pathogenesis of metabolic disease and type 2 diabetes mellitus. Lifestyle choices and genetic factors lead to persistent nutrient overload, overwhelming metabolic processes and leading to mitochondrial dysfunction and excessive ROS production. Supraphysiologic ROS levels activate inflammatory pathways through myokine/cytokine release and activation of transcription factors. This inflammatory response and damaging ROS molecules lead to an increase in misfolded proteins causing endoplasmic reticulum stress and further shifts in redox balance compounding oxidative distress. This activates stress pathways like JNK and inhibits impairs insulin signaling, reducing the ability of skeletal muscle to maintain nutrient homeostasis, further driving nutrient overload. Solid arrows depict the self-reinforcing cycle, while the dashed arrow indicated external factors that contribute to its initiation and amplification.

Skeletal muscle, normally reliant on tightly regulated redox cues, becomes vulnerable to imbalance as metabolic disease progresses. Chronic nutrient overload and dysregulated metabolism drive oxidative distress, with factors such as excess nutrient intake and sedentary behavior contributing to T2DM and other cardiometabolic pathologies. Hyperglycemia, advanced glycation, dyslipidemia, and low-grade inflammation collectively elevate ROS levels in skeletal muscle (Figure 2) [105]. Excessive mitochondrial ROS directly suppresses insulin signal transduction, promoting insulin resistance [105]. This oxidative burden can also overwhelm endogenous defenses and cause molecular damage, including increased protein carbonylation and lipid peroxidation byproducts [106,107].

Protein carbonylation involves the introduction of carbonyl groups (aldehydes and ketones) into proteins, often as a result of direct ROS attack or through secondary reactions with lipid peroxidation products. Elevated protein carbonyl content in skeletal muscle is a hallmark of oxidative distress in insulin-resistant states [106]. Specifically, acute overfeeding in healthy individuals can induce insulin resistance accompanied by oxidative distress and carbonylation of the GLUT4 glucose transporter [106]. Moreover, protein carbonylation is associated with loss of protein function, as it affects histidine, lysine, and cysteine residues located within enzyme active sites or at protein–protein interfaces [108].

Unlike physiological ROS signaling, lipid peroxidation is driven by hydroxyl radicals that attack polyunsaturated fatty acids (PUFAs) in membranes, initiating chain reactions that generate reactive aldehydes [107,108]. The most notable aldehydes include 4-hydroxy-2-nonenal (4-HNE) and 4-oxo-2-nonenal (4-ONE) from n-6 fatty acids, and 4-hydroxy-2-hexenal (4-HHE) from n-3 fatty acids [108]. These aldehydes can react with protein residues, leaving carbonyl groups attached and disrupting protein function [108]. In skeletal muscle of insulin-resistant individuals, elevated levels of 4-HNE protein adducts have been observed, correlating with glucose disposal rate, waist circumference, body mass index (BMI), and severity of insulin resistance [107]. 4-HNE has also been shown to covalently modify and inhibit Akt, leading to decreased GLUT4 function and glucose uptake [109]. Furthermore, experimental elevation of lipid peroxidation products directly impairs insulin action. Acute exposure to 4-HHE in muscle cells and rodents impairs insulin-stimulated glucose uptake and signaling, induces carbonylation, and depletes GSH [110]. Interestingly, boosting cellular GSH to detoxify 4-HHE prevents its associated insulin resistance, further underscoring the impact of lipid peroxidation on skeletal muscle insulin sensitivity [110]. It was recently shown in a mouse model of diabetes that muscle weakness and atrophy, and the accompanying ROS and lipid hydroperoxide elevation in diabetic skeletal muscle, could be protected against by supplementation with deuterium-enforced PUFAs, which are resistant to lipid peroxidation [111]. This work highlights lipid peroxidation as a potential treatment target for the protection of skeletal muscle in diabetes-associated muscle atrophy.

A major consequence of chronic oxidative distress in skeletal muscle is the disruption of normal insulin signaling. Under these conditions, excess ROS chronically activate stress-sensitive kinases, including p38 MAPK, JNK, NF-κB, and PKC, which shift from their physiological signaling roles to inhibitory functions by phosphorylating IRS-1 and IRS-2 [112,113,114,115,116,117,118]. This aberrant phosphorylation prevents proper tyrosine phosphorylation and accelerates IRS degradation [112], thereby blunting the insulin response. Notably, kinases such as PKC and JNK emerge repeatedly in metabolic disease progression, linking redox imbalance to impaired insulin signaling and inflammation.

### 3.2. Redox-Induced PTMs

Beyond oxidative damage, excessive ROS can disrupt insulin signaling by inducing inhibitory post-translational modifications. High ROS levels impair IR autophosphorylation and downstream PI3K/Akt activation through such modifications. For example, S-nitrosylation, the covalent attachment of an NO group to protein thiols, is markedly elevated in diabetic skeletal muscle [119]. Substantial S-nitrosylation of IR, IRS-1, and Akt has been observed in obese rodent models, inhibiting insulin signaling and promoting insulin resistance [119]. Increased S-nitrosylation has also been detected in human skeletal muscle with higher BMI, highlighting this pathway as a potential therapeutic target [119]. Furthermore, Akt can be reversibly inactivated by S-nitrosylation at key cysteine residues, further implicating this modification in the pathogenesis of insulin resistance [120].

Under oxidative and nitrosative distress, S-glutathionylation can also occur on cysteines, where a GSH molecule is added to form disulfide bonds; this modification has regulatory effects during metabolic disease progression [121]. In a pro-oxidative state, S-glutathionylation is a reversible process that occurs on many proteins in myocytes, serving to prevent oxidative damage and protect muscle contractile function [121]. However, excess S-glutathionylation may inhibit muscle fiber activity by impairing calcium uptake [121,122]. In particular, over-oxidation of the sarcoplasmic reticulum calcium-ATPase pump (SERCA) at a critical cysteine (Cys674) via S-glutathionylation (or other oxidative modifications) reduces its activity, slowing calcium reuptake into the sarcoplasmic reticulum [122]. This prolongs cytosolic calcium elevations after contraction and contributes to muscle fatigue and weakness under oxidative distress [122]. Taken together, these inhibitory post-translational modifications illustrate how redox imbalance not only disrupts insulin signaling but also compromises fundamental processes of muscle contractility and metabolism, laying the groundwork for broader mitochondrial dysfunction.

### 3.3. Mitochondrial Dysfunction

Mitochondria are a critical site where nutrient overload, redox imbalance, and impaired energy metabolism intersect in the development of insulin resistance. In the early stages of insulin resistance, nutrient excess provokes increased mitochondrial ROS production even before overt mitochondrial impairment is detectable [37]. Elevated fatty acid supply drives a higher electron flux through the ETC, which, when coupled with nutrient-induced uncoupling or electron leak, enhances O_2_^•−^ and H_2_O_2_ generation [37]. These ROS can damage mitochondrial DNA, respiratory chain proteins, and metabolic enzymes, progressively diminishing mitochondrial oxidative capacity.

A major component of this reduced capacity involves mitochondrial fatty acid oxidation, which becomes perturbed in nutrient-excess states. Paradoxically, obesity-related insulin resistance in muscle is often characterized by elevated β-oxidation flux that outpaces the tricarboxylic acid (TCA) cycle capacity [123,124]. This mitochondrial overload leads to incomplete fatty acid oxidation, evidenced by accumulation of partial β-oxidation intermediates and depletion of TCA cycle intermediates [123,124]. These intermediates, such as acylcarnitines, can directly contribute to insulin resistance, as they impair insulin signaling and induce metabolic inflexibility [124,125]. In both mice and human myocytes, experimental elevation of the long-chain acylcarnitine palmitoylcarnitine decreases glucose, induces hyperglycemia, and impairs Akt phosphorylation; conversely, reducing acylcarnitine accumulation improves insulin sensitivity [125].

Oxidative distress further worsens mitochondrial fatty acid oxidation efficiency. Reactive aldehydes from lipid peroxidation can inhibit β-oxidation enzymes; for example, in fatty liver disease a 4-HNE adduct on carnitine palmitoyltransferase 1 (CPT1) has been identified as a cause of impaired mitochondrial fatty acid uptake and oxidation [126]. By a similar mechanism in muscle, excess 4-HNE or related lipid aldehydes could diminish CPT1 activity and other metabolic enzymes, thereby promoting lipid accumulation. Nutrient oversupply thus establishes a cycle in which incomplete fat oxidation generates ROS, and ROS in turn hinders mitochondrial fat oxidation, amplifying insulin resistance. To better characterize this relationship in human skeletal muscle, Fiorenza et al. performed an in vivo study inducing lipid overload via intravenous lipid infusion [127]. They demonstrated that manipulating redox balance with the mitochondrially targeted antioxidant mitoquinone enhanced insulin-stimulated glucose uptake in skeletal muscle by augmenting GLUT4 translocation and reducing mitochondrial oxidative burden. This work directly links mitochondrial oxidant production to lipid overload-induced insulin resistance in human skeletal muscle.

Mitochondrial dynamics, or the balance between organelle fission and fusion, are also disrupted by oxidative distress. Mitochondrial fragmentation is frequently observed in skeletal muscle from obese and T2DM patients [128,129]. One mechanism involves elevated ROS and RNS shifting dynamics toward fission by post-translationally modifying fission and fusion proteins. For example, S-nitrosylation of GTPase dynamin-related protein 1 (DRP1) increases its oligomerization and promotes excessive mitochondrial fission under nitrosative distress [130]. This fragmentation is exacerbated by impaired mitochondrial fusion proteins: OPA1 and MFN2 expression is reduced in skeletal muscle from individuals with obesity and T2DM [128], and OPA1 itself can be oxidatively modified, with S-nitrosylated OPA1 detected in stressed tissues [130]. The net result is smaller, disconnected mitochondria that are less efficient at ATP production. Notably, mitochondrial fragmentation alone can impair insulin-stimulated Akt activity and glucose uptake in myocytes, underscoring its contribution to redox-driven insulin resistance [129,131].

### 3.4. Lipid Accumulation

Another defining feature of metabolic disease is ectopic lipid accumulation in non-adipose tissues, including skeletal muscle, where it directly contributes to redox imbalance and insulin resistance. When caloric intake is chronically high, adipose tissue storage capacity is exceeded, and surplus lipids infiltrate muscle fibers. In skeletal muscle, excess fat accumulates within myocytes as intramyocellular lipid droplets or in the interstitial spaces as extramyocellular adipose tissue [132,133]. Intramyocellular lipid accumulation is closely linked to insulin resistance, as it directly interferes with insulin signaling and reduces metabolic flexibility when lipid stores are overloaded [132]. Extramyocellular fat deposition, or myosteatosis, also correlates with muscle insulin resistance in both normal glycemic and diabetic individuals, though its influence is less direct than intramyocellular lipids [133]. Recently, fasting-induced lipid-mediated insulin resistance was studied in human vastus lateralis muscle to assess the impact of muscle fiber type on insulin resistance [134]; individuals with higher expression of type I muscle fibers exhibited a greater capacity to oxidize lipids and had worsened insulin sensitivity compared to individuals who had greater expression of type II muscle fiber despite similar levels of plasma lipids. This highlights the importance of considering muscle fiber type when assessing mechanisms of insulin resistance in skeletal muscle.

This lipid surplus, combined with reduced oxidative capacity, further induces ROS production that exacerbates insulin resistance in skeletal muscle [37]. Diacylglycerols (DAGs) and ceramides are two ectopic bioactive lipids known to accumulate in muscle and inhibit insulin signaling [135,136]. Elevated plasma free fatty acids drive intramyocellular DAG synthesis, which in turn activates canonical and non-canonical PKC pathways in skeletal muscle [135,137]. Activated PKC phosphorylates components of the insulin signaling pathway, decreasing IRS-1-associated PI3K activity and impairing Akt activation [135,138]. PKC activation by DAG also triggers pro-inflammatory pathways, as increased PKC activity promotes degradation of IκB-⍺, an inhibitor of NF-κB [135]. This allows NF-κB to translocate into the nucleus and induce inflammatory genes, thereby compounding local inflammation and oxidative distress.

Ceramides can activate phosphatases like protein phosphatase 2 A or PKC-Ζ disrupting Akt signaling, and can also promote mitochondrial apoptotic pathways and oxidative distress [136,139]. In excess, ceramides continually contribute to mitochondrial stress and further tip the redox balance toward an oxidative state in skeletal muscle [136]. It was recently demonstrated that this mitochondrial dysfunction is linked to ceramide-induced coenzyme Q (CoQ) deficiency in L6 myotubes, which causes a reduction in mitochondrial complex expression and subsequent mitochondrial dysfunction, oxidative distress, and insulin resistance [140]; insulin sensitivity was restored when either ceramide concentration was reduced or CoQ was supplemented in mice fed a high-fat diet (HFD). As well, sarcolemmal ceramide accumulation was recently associated with a rise in circulating oxidized phosphatidylcholine species, which can be found in low-density lipoprotein cholesterol [141]. Dyslipidemia associated with metabolic syndrome and T2DM helps to explain the increased ceramide levels in skeletal muscle that contribute to insulin resistance and muscle inflammation in T2DM. Interestingly, metabolomic assessment of plasma and skeletal muscle from HFD-fed mice revealed differential regulation of phosphatidylcholine abundance dependent on nuclear-mitochondrial genetic mismatch [142].

Together, these mechanisms illustrate how the lipotoxic environment in metabolic disorders provokes redox imbalance by both fueling ROS generation and activating stress and inflammatory signaling that blunt insulin action. Further, genetic factors influence HFD-associated metabolite patterns in skeletal muscle, helping to explain differential susceptibility to nutrient overload and subsequent metabolic disease development seen in human populations. Future work should consider how genetic susceptibility to metabolic diseases influences mechanisms driving insulin resistance, and how ‘omics technologies can be used to identify these distinct patterns.

### 3.5. Inflammation

Chronic low-grade inflammation is a central feature of obesity and T2DM, converging with redox distress to impair insulin sensitivity in skeletal muscle. Pro-inflammatory cytokines such as TNF-α, interleukin-6 (IL-6), and interleukin-1 (IL-1) are systemically elevated and enriched in insulin-sensitive tissues, partly due to immune cell infiltration and activation of macrophages or muscle fiber signaling pathways [143]. Mechanistically, pro-inflammatory cytokines activate inhibitory kinases and increase cellular ROS production [143]. For example, TNF-α interferes with insulin signaling by promoting serine phosphorylation of IRS-1 and reducing GLUT4 translocation [144,145]. TNF-α signaling also stimulates NOX activity and mitochondrial ROS emission, further compounding oxidative distress in muscle cells [145].

Clinically, obese patients with T2DM exhibit elevated IL-6 and TNF-α, which correlates with the severity of insulin resistance. For instance, IL-6 concentrations are significantly higher in obese individuals and show a positive correlation with homeostatic model assessment of insulin resistance [146]. Both diabetic and prediabetic patients also have higher TNF-α levels than their lean counterparts, which correlate strongly with increased insulin resistance [147]. This clinical evidence underscores the link between systemic inflammation and insulin resistance in skeletal muscle. Importantly, the relationship between inflammation and oxidative distress is bidirectional. ROS act as signaling molecules that activate redox-sensitive transcription factors such as NF-κB and AP-1; in excess, this upregulates pro-inflammatory genes including TNF-α [143]. This positive feedback loop means that initial metabolic stress provokes inflammation, which then amplifies oxidative distress, exacerbating insulin resistance and contributing to muscle fiber dysfunction, damage, or atrophy.

### 3.6. Antioxidant Defenses

As metabolic disease progresses, endogenous antioxidant defenses in skeletal muscle become increasingly insufficient to counterbalance supraphysiologic ROS levels. While muscle cells attempt to compensate through redox sinks such as upregulating GSH synthesis for S-glutathionylation, chronic nutrient overload can overwhelm these systems. Human studies have shown that GSH levels are significantly lower in severely obese individuals compared to normal-weight controls, consistent with rat models demonstrating decreased GSH within just two weeks of HFD administration [148,149,150]. Similarly, sustained oxidative load is associated with reduced expression or activity of SODs and GPXs [151,152]. When antioxidant defenses are depleted, redox-sensitive proteins remain oxidized for longer durations, and oxidative damage accumulates, further aggravating insulin resistance. Conversely, antioxidant supplementation in metabolic disease models has been shown to partially mitigate insulin resistance by restoring redox balance, underscoring the protective role of these systems [153]. Interestingly, compensatory mechanisms may also exist: in rat skeletal muscle, SOD2 deletion did not significantly impair whole-body metabolism, suggesting that GPX or PRX activity may increase to counteract the loss [154].

In contrast to the deleterious effects of nutrient excess, regular exercise triggers adaptive antioxidant responses in skeletal muscle highlighted by upregulation of antioxidant enzymes [155]. Moderate aerobic training increases SOD2, GPX1, and PRDX5 levels in skeletal muscle of patients with T2DM by 37–66% [156]. Endurance exercise similarly has been found to boost SOD in skeletal muscle of obese men [157]. Such exercise-induced enhancements in antioxidant capacity fortify muscle redox defenses, highlighting how physiological adaptations like training strengthen these systems, whereas chronic nutrient overload overwhelms them.

As discussed, NOX enzymes are crucial for modulating redox homeostasis and mediating skeletal muscle adaptation to exercise. Nox2 deficiency in mice exacerbates the impact of HFD on body weight, body composition, and glucose tolerance and inhibits several of the beneficial metabolic adaptations to exercise [158]; this work highlights Nox2 and its ROS contributing properties as a key mediator of exercise-induced benefits to skeletal muscle in the context of diet-induced obesity. Nox4 knockout compromises exercise capacity and antioxidant defenses, while promoting oxidative distress and insulin resistance in aging and diet-induced obesity conditions [50]. Nox4 was also recently highlighted a regulator of sexual dimorphic adaptations to lipid metabolism [159]. While Nox4 knockout in mice reduced adiposity, lipid accumulation, and improved glucose tolerance in male, HFD-fed mice, female mice exhibited the opposite effects when Nox4 was knocked out. As well, skeletal muscle gene expression showed unique patterns of gene expression in male and female mice related to redox-sensitive transcriptional networks that helped to shape sex-specific responses to HFD. This work showcases the important influence of sex on redox homeostasis in skeletal muscle.

Assessment of redox balance in skeletal muscle relies on several oxidative biomarkers, though not all demonstrate equal reliability. The glutathione redox ratio (GSH/GSSG) reflects cellular redox capacity with higher GSSG indicating greater oxidative distress [160]. While informative, GSH/GSSG is prone to assay artifacts and subsequent misinterpretation [160,161]. 4-HNE adducts and protein carbonyls indicate cumulative membrane damage and protein oxidation, respectively, but may miss transient redox changes due to their stability as end-products [162]. PRX’s undergo hyperoxidation in response to H_2_O_2_ flux which can be measured to identify ongoing or transient redox changes, but hyperoxidation assays can saturate under extreme distress and depend heavily on tissue handling techniques [163,164]. Antioxidant enzyme activities such as SOD/GPX can be assessed, though solo interpretation of changes may be misleading due to factors other than ROS burden impacting activity [165,166]. Given the variable reliability of individual assays, combining multiple markers and enforcing standardized protocols is recommended to enhance reliability and comparability. To address some of these challenges, Murphy et al. recently described guidelines for measuring ROS and oxidative damage in cells and in vivo to improve standardization across the field [167]. Advancements in redox measurement models in the context of metabolic diseases includes the development of a transgenic T2DM mouse model expressing redox-sensitive green fluorescent proteins that can distinguish between whole cell and mitochondrial ROS [168].

### 3.7. Endoplasmic Reticulum Stress

Another mechanism linking redox imbalance to metabolic dysfunction is endoplasmic reticulum (ER) stress. The ER is responsible for proper folding of secretory and membrane proteins, a process that requires an oxidizing environment supported by a network of chaperones and folding enzymes [169]. Under chronic caloric excess and insulin-resistant conditions, the protein-folding demand and accumulation of misfolded proteins can exceed the ER’s capacity, triggering the unfolded protein response (UPR) [169,170]. The adaptive UPR initially works to restore homeostasis, but if unresolved, it drives chronic activation ER stress pathways such as PERK-eIF2⍺, contributing to insulin resistance and cell death [169,170].

A key redox component of the UPR is ER oxidoreductin-1α (ERO1α), an ER-resident oxidase upregulated by the UPR transcription factor CHOP [169]. ERO1α promotes disulfide bond formation by continuously re-oxidizing protein disulfide isomerase (PDI); in doing so, it transfers electrons to O_2_ and generates H_2_O_2_ [169,171]. Chronic UPR activation, as observed in obesity and T2DM, can therefore lead to hyperactivation of ERO1α and PDI-mediated oxidative folding, resulting in an over-oxidized ER lumen [169,171,172]. This detrimental state not only produces excess ROS within the ER, but also disrupts calcium homeostasis [169,170]. For instance, hyperoxidizing conditions can sensitize inositol 1,4,5-trisphosphate receptors, promoting abnormal calcium release from the ER [173]. The leaked calcium subsequently overloads mitochondria, inducing additional oxidative distress and apoptosis [174]. Consistent with this model, metabolic stressors in skeletal muscle elevate ER stress and UPR markers, directly linking nutrient excess to this maladaptive pathway [175]. Sustained ER stress ultimately promotes insulin resistance, exemplified by activation of JNK, which phosphorylates IRS-1 and disrupts insulin signaling [172,176].

Taken together, these mechanisms (summarized in Table 2) demonstrate how redox imbalance permeates every level of skeletal muscle biology—damaging proteins and lipids, impairing organelle function, amplifying inflammatory pathways, and exhausting antioxidant defenses. The convergence of these processes creates a self-reinforcing cycle of oxidative distress and insulin resistance that propels the progression of metabolic disease. To mitigate insulin resistance associated with T2DM, numerous therapies both pharmacological and lifestyle-based are prescribed, often in combination. Due to the intimate linkage between redox balance and insulin sensitivity described above, many of these therapies directly and indirectly modulate redox homeostasis in skeletal muscle; however, few were designed to target redox homeostasis and do so secondarily through modulation of some of the key metabolic signaling pathways discussed. The main target of many T2DM pharmacological therapies is to correct the nutrient imbalance and overload cells face by lowering blood glucose levels. An overview of the therapies available to T2DM patients and their impacts on redox homeostasis and clinical outcomes is discussed below.

## 4. Treatment-Based Changes to Redox Signaling in Skeletal Muscle

**Figure 3:**
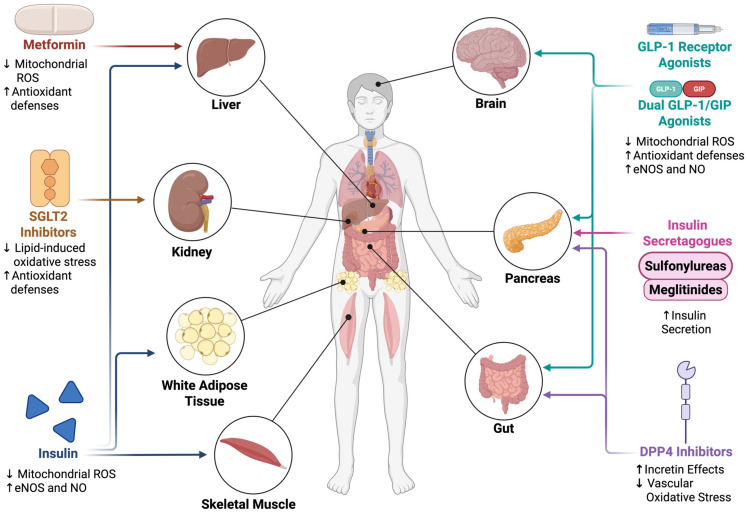
Systemic sites of action of type II diabetes therapies and their redox implications. Antidiabetic drug classes act across multiple tissues to regulate glucose homeostasis whilst influencing redox balance and ROS generation. Biguanides (metformin, dark red) act primarily on the liver and muscle to modulate Complex I activity, AMPK signaling, and antioxidant defenses. Insulin (blue) lowers glucose-induced oxidative distress and enhances NO-dependent vascular signaling. SGLT2 inhibitors (gold) act on the kidney but secondarily improve lipid handling, mitochondrial remodeling, and antioxidant capacity in skeletal muscle. Insulin secretagogues (sulfonylureas, meglitinides, pink) increase insulin secretion, indirectly affecting redox balance. Incretin-based therapies (GLP-1 receptor agonists, dual GLP-1/GIP agonists, and DPP-4 inhibitors, teal and lavender) exert multisystem effects, including improved mitochondrial biogenesis, reduced ROS production, and enhanced vascular perfusion. Arrows denote the primary organ(s) targeted by each therapy. Each class of drug’s mechanism of action on target organ(s) is/are discussed in depth in the main body text.

Therapeutic strategies for T2DM primarily target glycemic control but also exert wide-ranging effects on redox homeostasis in skeletal muscle. Several major drug classes, including biguanides, insulin, SGLT2 inhibitors, insulin secretagogues, incretin-based therapies (GLP-1 receptor agonists, dual GLP-1/GIP agonists, and DPP-4 inhibitors), demonstrate distinct, and often overlapping, influences on mitochondrial function, oxidative distress, and antioxidant signaling (Figure 3). For example, metformin reduces hepatic glucose production and modulates mitochondrial complex I activity and AMPK signaling; insulin blunts hyperglycemia-induced oxidative distress while engaging redox-sensitive pathways; and SGLT2 inhibitors, though renal in target, remodel skeletal muscle lipid composition and antioxidant defenses. Insulin secretagogues indirectly shape muscle redox balance through altered systemic insulin exposure, whereas incretin-based therapies act via mitochondrial biogenesis, antioxidant upregulation, and vascular mechanisms. An overview of these drug classes, their primary glucose-lowering actions, and known or proposed effects on skeletal muscle redox homeostasis is summarized in Table 3, which provides a framework for the more detailed mechanistic discussion that follows.

### 4.1. Biguanides (Metformin)

Biguanides are a class of antidiabetic agents that improve glycemic control primarily by reducing hepatic glucose production, decreasing intestinal glucose absorption, and enhancing peripheral glucose uptake [211]. Of this class, only metformin remains in clinical use and serves as the first-line therapy for T2DM [211]. While inhibition of mitochondrial complex I is often cited as a central mechanism, metformin’s actions vary with dose, duration, and tissue context, and extend to AMPK activation, modulation of mitochondrial energetics, and regulation of redox-sensitive transcriptional systems [177,212,213].

Mechanistically, metformin inhibits complex I of the mitochondrial ETC, limiting proton pumping and electron flux [212]. At therapeutic concentrations, this can reduce mitochondrial ROS production but higher doses or prolonged inhibition may paradoxically increase ROS generation and oxidative distress [214]. By constraining OXPHOS, pyruvate is increasingly diverted to lactate, lowering the NAD^+^:NADH ratio and reinforcing a reductive redox state [178]. These changes oppose oxidative distress in skeletal muscle and activate compensatory redox signaling pathways.

In vitro studies using cultured human myotubes demonstrate that metformin treatment induces dose-dependent increases in lactate secretion and glucose uptake, accompanied by a reduced ratio of oxygen consumption rate to extracellular acidification rate, reflecting a metabolic switch from OXPHOS to glycolysis [178]. In vivo, animal studies further show that metformin impairs ADP-stimulated mitochondrial respiration in both high- and low-capacity running rats, with reversibility depending on intrinsic aerobic fitness [179]. These findings suggest that baseline mitochondrial capacity dictates the extent of redox resilience to metformin.

A central mediator of this adaptation is AMPK, a redox-sensitive hub activated by shifts in energy charge and oxidative cues. Evidence from both cell culture and rodent models indicates that AMPK activation enhances fatty acid oxidation and improves coupling efficiency, thereby reducing excessive mitochondrial ROS [181]. Through AMPK signaling, metformin induces peroxisome proliferator-activated receptor gamma coactivator-1α (PGC-1α) and Nrf2 pathways, upregulating antioxidant enzymes (SOD, catalase, GPX) and supporting mitochondrial remodeling [180,181]. At the same time, AMPK-FoxO3a-HDAC6 signaling can increase myostatin expression, linking redox stress to muscle atrophy [182].

Clinically, metformin consistently improves glycemic control and insulin sensitivity, but direct evidence for skeletal muscle redox modulation in humans is limited. While preclinical studies demonstrate reduced mitochondrial ROS and activation of AMPK-Nrf2 antioxidant signaling [215,216], human biopsy studies primarily show metabolic adaptations without direct redox assessment [217]. Improvements in systemic oxidative distress markers have been reported, yet these reflect whole-body rather than muscle specific effects. Overall, metformin’s effects in skeletal muscle are context-dependent: at moderate doses it dampens mitochondrial ROS and promotes antioxidant defenses, while in settings of energetic vulnerability it may exacerbate oxidative distress and muscle loss.

### 4.2. Insulin

Insulin therapy is essential in type I diabetes due to autoimmune destruction of β-cells and in later-stage T2DM when β-cell failure leads to insufficient endogenous insulin production [93,183]. In skeletal muscle, insulin binding to its receptor activates the PI3K-Akt signaling cascade, promoting GLUT4 translocation and glucose uptake while simultaneously engaging redox-sensitive pathways [113].

In vitro and animal studies show that by lowering circulating glucose, insulin mitigates hyperglycemia-induced oxidative distress through multiple mechanisms: reducing mitochondrial substrate overload and electron leak, limiting ROS production from excess glucose oxidation, and decreasing advanced glycation end product (AGE) formation and signaling [218]. Within the insulin signaling pathway, Akt phosphorylates NOS at Ser1177, enhancing its activity and modulating NO signaling to promote vasodilation, enhanced perfusion, and mitochondrial oxidative efficiency while simultaneously reducing ROS-mediated NO scavenging. Acute insulin exposure can also suppress NOX activity and downregulate NF-κB signaling, decreasing cytosolic O_2_^•−^ production and reducing inflammatory cytokine expression, respectively. Together, these effects establish a more balanced redox environment in muscle and promote insulin sensitivity.

Despite this clear mechanistic rationale, direct evidence for redox modulation by exogenous insulin therapy in skeletal muscle remains limited, with most recent research emphasizing alternative pharmacotherapies and antioxidant strategies to restore redox balance [67,92,113].

### 4.3. Sodium-Glucose Cotransporter-2 (SGLT2) Inhibitors

Sodium-glucose cotransporter-2 (SGLT2) inhibitors lower plasma glucose by blocking renal glucose reabsorption in the proximal convoluted tubule of the kidneys, producing glucosuria independent of insulin secretion or sensitivity [184,219]. In addition to glycemic control, they confer cardiovascular and renal protection and can modestly reduce body weight [220]. Although their primary mechanism of action is renal, evidence indicates they also modulate skeletal muscle metabolism and redox signaling. By lowering circulating glucose and free fatty acids, SGLT2 inhibitors reduce mitochondrial substrate overload and electron leak in muscle fibers, indirectly diminishing ROS generation. Beyond these systemic effects, both preclinical and clinical studies support direct modulation of muscle mitochondrial signaling and redox-sensitive transcriptional networks.

In *db/db* mice, luseogliflozin improved skeletal muscle redox homeostasis by remodeling lipid composition, reducing harmful saturated fatty acids like palmitate and increasing protective monounsaturated fatty acids such as oleate [185]. This shift alleviates mitochondrial dysfunction, attenuates ER stress, and reduces lipid peroxidation. Luseogliflozin also downregulated muscle atrophy-related genes (FoxO1, myostatin, muscle-specific ubiquitin ligases) and decreased the accumulation of AGEs, consistent with reduced mitochondrial oxidative distress and proteolytic signaling. Suppression of elevated SOD2 and ETC complex expression further indicated relief of mitochondrial redox stress [186]. Similarly, in a 52-week clinical study in moderately obese Japanese patients with T2DM, luseogliflozin decreased fat mass while preserving skeletal muscle and bone, aligning with preclinical findings and supporting improved redox balance [187].

Similar effects are observed with other SGLT2 inhibitors. In HFD/streptozotocin rats, dapagliflozin promoted mitochondrial biogenesis via activation of the AMPK-PGC-1α-TFAM axis, enhancing OXPHOS efficiency and limiting electron leak. Dapagliflozin also improved mitochondrial fission/fusion dynamics by upregulating fusion proteins OPA1 and MFN2, and downregulating fission protein DRP1, thereby reducing ROS production and improving mitochondrial quality control. Collectively, these adaptations reduced oxidative distress, restored redox homeostasis, and improved insulin sensitivity in diabetic skeletal muscle [188]. Recent clinical evidence demonstrates that 6 weeks of dapagliflozin treatment in adults with T2DM increases skeletal muscle fatty acid uptake and oxidation, enhances ketone body utilization, and stimulates mitochondrial biogenesis. This shift in substrate preferences supports improved mitochondrial efficiency and redox balance. Similarly, in diet-induced obese mice, empagliflozin increased the expression of PGC-1α, Nrf1, and TFAM, accompanied by greater citrate synthase activity and improved subsarcolemmal and intermyofibrillar mitochondrial morphology, indicative of enhanced mitochondrial remodeling, redox balance, and metabolic flexibility in skeletal muscle [189].

Overall, SGLT2 inhibitors preserve skeletal muscle redox homeostasis by reducing lipid-induced oxidative distress, enhancing mitochondrial quality control, and supporting antioxidant defenses, with benefits observed across animal models and clinical studies.

### 4.4. Insulin Secretagogues

Insulin secretagogues stimulate pancreatic β-cells to release insulin by targeting ATP-sensitive potassium (K_ATP_) channels [221]. While effective at lowering blood glucose, their redox impact in skeletal muscle is largely indirect, mediated through altered insulin exposure and the risks of hypoglycemia.

#### 4.4.1. Sulfonylureas

Sulfonylureas, including glimepiride, glipizide, and gliclazide, act by closing K_ATP_ channels on β-cells, causing membrane depolarization, calcium influx, and insulin secretion independent of glucose concentration. This mechanism increases hypoglycemia risk, particularly with longer-acting agents [190]. In skeletal muscle, sulfonylureas primarily enhance insulin-stimulated glucose uptake, but direct redox effects appear limited. Clinical evidence links sulfonylurea use, particularly glibenclamide, to increased risk of muscle atrophy and reduced strength, potentially mediated by hypoglycemia-induced apoptosis and autophagy [191,192]. Additional reports suggest possible modulation of muscle K_ATP_ channels with implications for contractility and fatigue resistance, though tissue-specific subunit expression constrains these effects [191].

#### 4.4.2. Meglitinide Analogs

Meglitinide analogs, such as repaglinide and nateglinide, also target β-cell K_ATP_ channels but induce rapid, meal-dependent insulin secretion [222]. Their short action reduces the risk of prolonged hypoglycemia compared to sulfonylureas, thereby reducing the likelihood of hypoglycemia-associated redox imbalance. However, direct effects on skeletal muscle metabolism and redox homeostasis remain largely unexplored.

Together, these findings indicate that insulin secretagogues influence skeletal muscle redox balance largely through systemic changes in insulin exposure and hypoglycemia risk, with limited evidence for direct effects on muscle metabolism. Further in vivo studies examining redox-sensitive endpoints (ROS markers, SOD activity, mitochondrial dynamics) would be necessary to clarify whether these agents exert skeletal muscle-specific redox effects.

### 4.5. GLP-1 Receptor Agonists and Dual GLP-1/GIP Incretin Therapies

Glucagon-like peptide-1 receptor agonists (GLP-1 RAs), such as semaglutide, liraglutide, and exenatide, mimic endogenous GLP-1, activating the GLP-1 receptor to enhance insulin secretion, suppress glucagon, slow gastric emptying, and reduce appetite [223,224,225]. Beyond glucose-lowering, large randomized clinical trials demonstrate major benefits for cardiovascular protection and weight loss [226,227,228,229,230]. For example, SUSTAIN-6 reported a 26% reduction in cardiovascular events with semaglutide [227], while STEP 1 showed nearly 15% body weight loss with semaglutide plus lifestyle intervention [229]. These outcomes, together with strong glycemic efficacy, establish GLP-1 RAs as an emerging, leading therapy in T2DM management [231,232]. As part of their multisystem mechanism of action, GLP-1 RAs modulate redox balance across metabolic tissues, although direct evidence in skeletal muscle is limited [193,233]. Emerging studies nonetheless suggest they influence redox-sensitive signaling pathways, mitochondrial function, and inflammation in ways relevant to muscle health [234,235].

Dual GLP-1/GIP (glucose-dependent insulinotropic polypeptide) RAs such as tirzepatide activate both GLP-1 and GIP incretin receptors to further enhance insulin secretion, glucagon suppression, and appetite control [25,236,237]. The SURPASS clinical trials demonstrated the superior glycemic and weight loss efficacy of dual GLP-1/GIP RAs compared to GLP-1 RAs alone [224]. Mechanistically, these dual agonists are expected to impact redox homeostasis through many of the same pathways as GLP-1 RAs, though direct data in skeletal muscle remain scarce [205]. Preclinical evidence in C57BL/6 mice, KKAy mice, and Sprague-Dawley rats indicates that GLP-1 RAs and dual GLP-1/GIP agonists improve redox signaling in skeletal muscle and other tissues by enhancing mitochondrial quality control and antioxidant defenses [194,195,196,197]. Similarly, in these and other rodent models of T2DM, including Spontaneously Diabetic Torii (SDT) fatty rats, these drugs increase mitochondrial number and area, preserve key enzymes such as cytochrome c and citrate synthase, and improve mitochondrial morphology, resulting in greater oxidative efficiency and reduced ROS production [198,199,200,201,233],

Mechanistically, activation of the GLP-1 receptor stimulates Gαs-coupled adenylate cyclase and increases cAMP, leading to PKA activation. PKA phosphorylates DRP1 (at Ser637), suppressing excessive fission, while promoting fusion proteins OPA1 and MFN1/2, thereby maintaining a healthy mitochondrial network [238,239,240,241,242]. PKA and related kinases also regulate the mitochondrial calcium uniporter, boosting calcium-dependent dehydrogenase activity, optimizing substrate flux into the ETC, and reducing electron leak [243,244,245]. These adaptations preserve ATP synthesis while lowering ROS production. GLP-1 receptor signaling also engages PI3K/Akt and AMPK pathways, which enhance glucose uptake, stimulate mitophagy through PINK1/Parkin, and promote mitochondrial biogenesis via PGC-1α and Nrf1/2 [246,247,248,249,250,251,252,253]. Through these transcriptional programs, GLP-1 RAs upregulate antioxidant enzymes, including SOD, catalase, and glutathione systems [254,255,256].

In primary normal human coronary artery endothelial cells, GLP-1 receptor activation was shown to enhance eNOS phosphorylation via PI3K/Akt, increasing NO production [202,203,204,231]. NO improves perfusion, but also directly reduces O_2_^•−^ formation by NOX enzymes and stimulates antioxidant defenses [54,257,258,259]. These vascular effects further contribute to skeletal muscle redox balance. Dual GLP-1/GIP agonists act through parallel mechanisms at the GIP receptor, also activating cAMP-PKA signaling, DRP1 inhibition, and fusion protein upregulation, while converging on AMPK and PI3K/Akt pathways [252,253,254,255,256,260,261]. These signals reinforce mitochondrial biogenesis and antioxidant defenses, supporting a homeostatic redox state. Although direct skeletal muscle studies are limited, mechanistic evidence strongly suggests that dual agonists enhance redox resilience alongside improvements in metabolic efficiency.

Together, these findings position incretin-based therapies as promising modulators of skeletal muscle redox balance, although direct human evidence remains limited and further studies are needed to translate preclinical mechanisms into clinical insight.

### 4.6. DPP-4 Inhibitors

Dipeptidyl Peptidase-4 (DPP-4) inhibitors such as lingliptin, sitagliptin, saxagliptin, and alogliptin, act by blocking the DPP-4 enzyme responsible for degrading the incretin hormones GLP-1 and GIP [262]. In doing so, DPP-4 inhibitors prolong the action of these hormones, enhancing glucose-dependent insulin secretion while decreasing glucagon secretion [262,263]. This mechanism lowers blood glucose levels with a comparatively modest effect, making DPP-4 inhibitors generally weight-neutral and associated with a lower risk of hypoglycemia than more potent incretin-based therapies such as GLP-1 RAs [262,263,264]. Very little direct research has examined the effects of DPP-4 inhibition on redox homeostasis in skeletal muscle. However, by prolonging the actions of incretins such as GIP and GLP-1, DPP-4 inhibitors are expected to engage many of the same redox-protective mechanisms described for incretin-based therapies [262,263]. Beyond incretin effects, DPP-4 inhibitors also demonstrate intrinsic antioxidant properties. In n2-streptozotocin diabetic rats, treatment reduced NO levels in serum and pancreatic tissue in a dose-dependent manner [206]. Other work has linked DPP-4 inhibition to decreased vascular oxidative distress through suppression of inflammation and improved endothelial function [265].

Skeletal muscle itself expresses and releases DPP-4, where it regulates vasoactive peptides such as GLP-1 and neuropeptide-Y. Inhibiting DPP-4 increases GLP-1 bioactivity, activating eNOS and elevating NO production to promote vasodilation [54,204,231,257]. Improved vasodilation enhances perfusion and substrate delivery to myocytes while facilitating clearance of ROS-generating metabolites such as lactate [266,267]. However, clinical findings remain inconsistent: sitagliptin and alogliptin reduced NO-dependent vasodilation in some short-term studies [207,268], showed no effect in others [208,268], and improved vasodilation in trials with sitagliptin and vildagliptin [209,210,268].

Taken together, DPP-4 inhibitors may confer modest but variable benefits for redox balance in skeletal muscle, acting through both incretin-dependent pathways and vascular mechanisms that enhance O_2_ and nutrient supply while promoting clearance of oxidative by-products.

### 4.7. Summary of Therapuetic Effects on Skeletal Muscle Redox Signaling

While current T2DM therapeutics undoubtedly impact cellular redox state, specific studies in skeletal muscle are somewhat variable depending on the class of drug in question. Table 4 summarizes mechanisms of drug action that have demonstrated redox-modulating effects, but many of these are indirect, acting through blunting of nutrient overload, improvements in mitochondrial function, and modulation of the insulin signaling cascade without direct redox targets. Increased exploration of therapies that directly target skeletal muscle redox mechanisms is warranted to improve management of complex metabolic diseases like T2DM. For example, the fungal depsidone metabolite nidulin promotes glucose uptake in L6 myotubules regardless of muscle insulin sensitivity, through increased cytosolic H_2_O_2_ and calcium levels, as well as actions on key insulin signaling proteins [269]. This work demonstrates the utility of targeting skeletal muscle redox homeostasis as a promising avenue for antidiabetic strategies. Further work in this area may better target skeletal muscle metabolic dysfunction and improve standard-of-care for metabolic diseases.

## 5. Future Perspectives

This review highlights the crucial role of redox homeostasis in maintaining skeletal muscle health and demonstrates the vast body of evidence implicating redox-sensitive systems in the development of insulin resistance, subsequent metabolic syndrome, and T2DM. Despite this, current therapeutics for T2DM do not directly target these systems and lack focus on mediating skeletal muscle redox homeostasis. As well, many of the studies investigating redox markers are performed in preclinical models, with RCTs often overlooking therapeutic impact on muscle metabolism and redox homeostasis while sometimes favoring functional outcomes like lean mass and strength metrics. More research in mature muscles under in vivo conditions and the use of biosensors targeted to specific subcellular locations could better characterize redox sources and targets during metabolic disease progression and treatment. While not reviewed in depth herein, redox homeostasis in skeletal muscle is essential for mediating contraction and adaptation to exercise—directly implicating redox balance in outcomes related to lifestyle changes prescribed concomitantly with the discussed pharmacological therapeutics. Focus on skeletal muscle molecular outcomes in human participants may aid in increasing exercise tolerance in T2DM patients and, therefore, better adherence to sustainable treatment for management of metabolic diseases. While implementing redox-focused markers into clinical trials is important for deepening our understanding of redox homeostasis, such that these pathways can be leveraged for therapeutic benefit, we recognize the variable reliability of redox-sensitive biomarkers. We also suggest that better standardization in the field of redox biology will be essential for both reproducibility of results and reliability of findings. This variability may also be addressed through the development of redox-related biomarkers identified via ‘omics technology; this growing field has demonstrated promise in biomarker development for numerous diseases [270,271,272] and may serve as a promising avenue of biomarker development for skeletal muscle health in metabolic diseases [142]. As well, the impact of nutritional strategies and antioxidant supplementation deserves further research to not only identify the impact of these nutraceutical strategies but to better understand how these changes interact with current standard-of-care therapeutics.

## 6. Conclusions

Redox imbalance in skeletal muscle plays a central role in the pathogenesis of metabolic syndrome and T2DM. Chronic nutrient overload, mitochondrial dysfunction, lipid accumulation, inflammation, and ER stress converge to overwhelm antioxidant defenses and impair skeletal muscle signaling. This review emphasizes how oxidative and nitrosative distress disrupt insulin signaling through protein and lipid oxidation, mitochondrial fragmentation, and inflammatory pathways, ultimately driving muscle dysfunction and insulin resistance. Once this redox imbalance is established, it enters a self-reinforcing cycle that continuously amplifies itself through positive feedback loops. Current pharmacotherapies, like metformin and insulin therapy, directly target oxidative pathways to reduce distress, while others, like SGLT2, GLP1, and DPP4, act downstream on insulin signaling and metabolism to maintain a redox balance. While animal and cell models demonstrate strong redox effects, further investigations of these therapies in clinical studies are needed. Future progress will depend on integrating these redox biomarkers into clinical studies and optimizing treatment strategies both alone and in combination with standard therapies or lifestyle interventions. To strengthen translation to clinical trials, we recommend the following actionable measures: (i) harmonize redox biomarker assays across laboratories to allow cross-study comparisons, (ii) implement small, mechanistic phase II studies using skeletal muscle biopsies before and after exercise intervention to directly measure redox effects, (iii) systematically stratify participants by sex and age to uncover differential responses, and (iv) design trials testing pharmacological agents together with structured lifestyle programs to assess additive or synergistic benefits. These targeted strategies will provide funders and study designers with actionable guidance for incorporating redox endpoints in metabolic disease research. Recognizing restoration of redox homeostasis as both a therapeutic goal and a mechanistic framework may advance our understanding and treatment of metabolic disease and diabetes progression.

## Figures and Tables

**Table 2:** Redox modifications and their effects on skeletal muscle during metabolic disease progression.

Redox Modification	Mechanism	Action on Skeletal Muscle	Reference(s)
Protein carbonylation	Oxidation of amino acid side chains to introduce carbonyl groups	Loss of enzyme activity and structural protein function; carbonylation of GLUT4 impairs activity	[106,108]
Lipid peroxidation	Hydroxyl radical attack on PUFAs that generate aldehydes (4-HNE, 4-ONE, 4-HHE)	Akt2 modification, inhibition → reduced GLUT4 function and glucose uptake; increased carbonylation and GSH depletion	[107,108,109,110]
S-nitrosylation	Covalent atachment of NO to protein thiols	Modification of IR, IRS, Akt inhibiting insulin signaling; Akt/PKB inactivation	[119,120]
S-glutathionylation	Reversible addition of GSH to cysteine residues under oxidative distress	Excess modification impairs Ca^2+^ uptake into SR via SERCA inhibition → muscle fatigue	[121,122]
Mitochondrial ROS production	Nutrient overload → higher electron flux through ETC → enhanced ROS generation	mtDNA and respiratory chain damage → reduced oxidative capacity; incomplete fatty acid oxidation with acylcarnitine accumulation → impaired Akt phosphorylation	[37,123,124,125]
Lipid accumulation	Ectopic lipid (DAGs, ceramides) deposition in myocytes during caloric surplus	DAG synthesis → PKC activation → decreased IRS-1 activity, impaired Akt activation, increased NF-κB activity; Ceramide activation of phosphatases → Akt dephosphorylation and apoptosis; Ceramide-induced depletion of CoQ → reduced mitochondrial respiratory complexes → mitochondrial dysfunction and oxidant production	[132,133,135,136,137,138,139,140]
Antioxidant defense depletion	Oxidative overload → reduced SOD and GPX activity	Over-oxidation of redox-sensitive proteins → accumulation of oxidative damage → insulin resistance	[151,152,153]

**Table 3:** T2DM pharmacotherapies and their effects on skeletal muscle redox homeostasis.

Drug Class	Primary Glucose-Lowering Mechanism	Known/Proposed Effects on Redox Homeostasis in Skeletal Muscle	Types of Evidence	References
Biguanides (Metformin)	↓ hepatic glucose production, intestinal glucose absorption ↑ peripheral glucose uptake	Modulates complex I and AMPK activation ↓ mitochondrial ROS ↑ antioxidant defenses	In vitro—isolated rat liver mitochondria; C2C12 myoblasts; primary human myotubes. In vivo—HCR/LCR rats; *ob/ob* mice; *db/db* mice.	[177,178,179,180,181,182]
Insulin	Activates insulin receptor signaling (PI3K-Akt) to ↑ GLUT4-mediated uptake	Mitigates hyperglycemia-induced oxidative distress ↓ mitochondrial ROS and AGEs ↑ eNOS-mediated NO signaling	In vitro—C2C12 myoblasts; primary human myotubes. Indirect—RCT; animal models.	[67,92,183]
SGLT2 Inhibitors	Inhibit renal SGLT2 transporter in proximal tubules	↓ lipid-induced oxidative distress ↑ mitochondrial quality/antioxidant defenses	In vivo—STZ/HFD rats; *db/db* mice. Ex vivo—skeletal muscle fibers. Indirect—cohort study, cross-over study.	[184,185,186,187,188,189]
Insulin Secretagogues	Stimulate insulin secretion by closing β-cell K_ATP_ channels	Indirect through changes in systemic insulin and glucose	In vivo—Kir6.1[V65M] mice. Indirect—cohort study.	[190,191,192]
GLP-1 Receptor Agonists Dual GLP-1/GIP Agonists	Activate GLP-1 and GIP receptors ⟶ increased insulin, ↓ glucagon, ↓ gastric emptying, ↓ appetite	↑ Mitochondrial biogenesis, mitophagy, mitochondrial fusion, antioxidant expression ↑ Gαs-coupled adenylate cyclase stimulation ⟶ increased cAMP ⟶ increased PKA activation ⟶ increased calcium-dependent dehydrogenase activity ⟶ increased substrate flux ↓ mitochondrial electron leak ⟶ decreased ROS ↑ eNOS ⟶ NO ⟶ improved perfusion ↓NOX-mediated ROS production	In vivo—C57BL/6, KKAy mice; *ob/ob* mice; Sprague-Dawley rats; albino rats. In vitro—mouse hepatocytes. Indirect—RCT; cohort study; cross sectional study.	[193,194,195,196,197,198,199,200,201,202,203,204,205]
DPP-4 Inhibitors	Inhibit DPP-4 enzyme ⟶ prolong GLP-1 and GIP activity, ↑ insulin, ↓ glucagon	↑ Incretin-mediated responses Direct antioxidant and vascular effects (↓ NO, ↓ vascular oxidative distress, ↑ perfusion)	In vivo—STZ rats. Indirect—cohort study; RCT(s); cross-over study.	[206,207,208,209,210]

↑-increase; ↓ decrease; ⟶ indicates sequence of events.

**Table 4:** Summary of redox-specific mechanisms demonstrated by common T2DM pharmacological therapies.

Mechanism of Action of Redox Impacts	T2DM Drug Classes	References
Inhibition of mitochondrial complex I	Biguanides	[177]
PI3K-Akt signaling	Insulin Incretin Therapies	[113,204,246]
AMPK-PGC-1α	Biguanides SGLT2 Inhibitors Incretin therapies	[180,181,182,231]
Enhancement of mitochondrial quality (dynamics, mitophagy, etc.)	SGLT2 Inhibitors Incretin therapies	[189,194,195,196,197,198,199,200,201,233]
Regulation of vascular tone through NO signaling	Insulin Incretin therapies	[202,203,204,218,231]
Reduction in substrate overload and lipid-induced oxidative distress	Biguanides SGLT2 Inhibitors	[181,185]
Inhibition of β-cell K_ATP_ Channels	Insulin Secretagogues	[191,221,222]

## Data Availability

Not applicable.

## Abbreviations

The following abbreviations are used in this manuscript:

4-HHE	4-hydroxy-2-hexenal
4-HNE	4-hydroxy-2-nonenal
4-ONE	4-oxo-2-nonenal
AGE(s)	Advanced glycation end-product(s)
Akt	Protein kinase B
AMPK	AMP-activated kinase(s)
AP-1	Activator protein 1
CPT1	Carnitine palmitoyltransferase 1
CVD	Cardiovascular disease
COX(s)	Cyclooxygenase(s)
DAG(s)	Diacylglycerol(s)
DPP-4	Dipeptidyl peptidase-4
DRP1	Dynamin-related protein 1
ER	Endoplasmic reticulum
ERO1α	Oxidoreductin-1α
ETC	Electron transport chain
FOXO	Forkhead box class O
GIP	Glucose-dependent insulinotropic polypeptide
GLP-1	Glucagon-like peptide-1
GLP-1RAs	Glucagon-like peptide-1 receptor agonist(s)
GLUT4	Glucose transporter 4
GPX(s)	Glutathione peroxidase(s)
GSH/GSSG	Glutathione (reduced/oxidized)
GSK3	Glycogen synthase kinase 3
H_2_O_2_	Hydrogen peroxide
HFD	High-fat diet
IL	Interleukin
IR	Insulin receptor
IRS	Insulin receptor substrate(s)
JNK	c-Jun N-terminal kinase
LOX(s)	Lipoxygenase(s)
MAPK	Mitogen-activated kinase
MFN1/2	Mitofusin 1/2
mTORC1	Mammalian target of rapamycin complex 1
NADH/NAD^+^	Nicotinamide adenine dinucleotide (reduced/oxidized)
NADPH	Nicotinamide adenine dinucleotide phosphate
NF-κB	Nuclear factor kappa-light-chain-enhancer of activated B cells
NO	Nitric oxide
(e)(i)(n)NOS	(endothelial) (inducible) (neuronal) nitric oxide synthase
NOX	NAD(P)H oxidase
Nrf2	Nuclear factor erythroid 2-related factor
O_2_^•−^	Superoxide
ONOO^−^	Peroxynitrite
OPA1	Optic atrophy 1 (protein)
OXPHOS	Oxidative phosphorylation
PDI	Protein disulfide isomerase
PDK1	Phosphoinositide-dependent kinase 1
PGC-1α	peroxisome proliferator-activated receptor gamma coactivator-1α
PI3K	Phosphoinositide 3-kinase
PIP_2_	Phosphatidylinositol bisphosphate
PIP_3_	Phosphatidinositol-3,4,5-triphosphate
PKA	Protein kinase A
PKC	Protein kinase C
PLA2	Phospholipase A
PRX(s)	Peroxiredoxin(s)
PUFA(s)	Polyunsaturated fatty acid(s)
Rac1	RAS-related C3 botulinum toxin substrate 1
Redox	Oxidation-reduction
RNS	Reactive nitrogen species
ROS	Reactive oxygen species
SERCA	Sarco/endoplasmic reticulum calcium ATPase
SGLT2	Sodium-glucose transport protein 2
SOD	Superoxide dismutase
T2DM	Type 2 diabetes mellitus
TCA	Tricarboxylic acid (cycle)
TFAM	Transcription factor α mitochondrial
TNF	Tumor necrosis factor
UPR	Unfolded protein response
XOR	Xanthine oxidoreductase
